# Corrigendum: poreCov - An Easy to Use, Fast, and Robust Workflow for SARS CoV-2 Genome Reconstruction *via* Nanopore Sequencing

**DOI:** 10.3389/fgene.2022.875644

**Published:** 2022-03-14

**Authors:** Christian Brandt, Sebastian Krautwurst, Riccardo Spott, Mara Lohde, Mateusz Jundzill, Mike Marquet, Martin Hölzer

**Affiliations:** ^1^ Institute for Infectious Diseases and Infection Control, Jena University Hospital, Jena, Germany; ^2^ RNA Bioinformatics and High-Throughput Analysis, Friedrich Schiller University Jena, Jena, Germany; ^3^ Methodology and Research Infrastructure, MF1 Bioinformatics, Robert Koch Institute, Berlin, Germany

**Keywords:** Nextflow, bioinformatics, coronavirus – COVID-19, lineages, docker, SARS-CoV-2, pipeline, nanopore sequencing

In the original article, we neglected to include the funder “Ministry for Economics, Sciences and Digital Society of Thuringia (TMWWDG), DigLeben-5575/10-9 to Sebastian Krautwurst and Mara Lohde”. A correction has been made to the the funding section:

“This work received financial support from the Ministry for Economics, Sciences and Digital Society of Thuringia (TMWWDG), under the framework of the Landesprogramm ProDigital (DigLeben-5575/10-9) and by grants from the Federal Ministry of Education and Research (Grant No. 01KX2021).”

The authors apologize for this error and state that this does not change the scientific conclusions of the article in any way. The original article has been updated.

